# Case Report: Fundus findings in myelin oligodendrocyte glycoprotein-associated optic neuritis

**DOI:** 10.3389/fopht.2025.1620614

**Published:** 2025-09-22

**Authors:** John R. Wilson, Olivia W. Cummings, Matthew S. Elitt, Gregory P. Van Stavern

**Affiliations:** John F. Hardesty Department of Ophthalmology and Visual Sciences, Washington University in St.Louis School of Medicine, St. Louis, MO, United States

**Keywords:** myelin oligodendrocyte glycoprotein-associated optic neuritis, MOGAD, Roth spots, retinal hemorrhage, atypical optic neuritis, peripapillary hemorrhage, acute vision loss

## Abstract

Myelin oligodendrocyte glycoprotein-associated optic neuritis (MOG-ON) is a sight-threatening demyelinating disorder that can present with various ocular manifestations. Here, we describe a unique case of bilateral MOG-ON with unilateral retinal hemorrhages and Roth spots. We present the case of a 48-year-old man with acute-onset painful, severe vision loss in both eyes. Initial fundoscopic examination revealed bilateral optic nerve edema with unilateral retinal hemorrhages and Roth spots. Imaging was notable for perineural enhancement along both optic nerves. Serological testing revealed elevated MOG antibodies. The patient was treated with high-dose intravenous steroids followed by plasmapheresis, which resulted in substantial clinical improvement. We conducted a literature review of all available studies published before March 30, 2025, using PubMed, including the keywords “myelin oligodendrocyte glycoprotein-associated optic neuritis,” “myelin oligodendrocyte glycoprotein,” “optic neuritis,” “Roth spots,” and “retinal hemorrhage.” We found that this is the first reported case in a male patient—and only the third reported case overall—of retinal hemorrhages and Roth spots occurring in the context of MOG-ON. While retinal hemorrhage and Roth spots have not historically been associated with MOG-ON, recognizing the spectrum of fundoscopic findings is crucial for the early diagnosis and management of this potentially sight-threatening disease.

## 1 Introduction

Myelin oligodendrocyte glycoprotein (MOG) is essential for the myelination of the nerves within the central nervous system. Antibodies against MOG are a recognized cause of optic neuritis. We report a case of bilateral MOG-associated optic neuritis (MOG-ON) presenting with unilateral peripheral retinal hemorrhages and Roth spots. A 48-year-old man was referred to our academic center for further evaluation of severe, painful bilateral vision loss. His medical history was notable for hereditary hearing loss, dyslipidemia, and well-controlled hypertension. His ocular history was unremarkable, except for color vision deficiency. Written consent to publish this case report was obtained from the patient.

## 2 Case presentation

Prior to the development of vision loss, the patient had an upper respiratory infection and was prescribed methylprednisolone and a 6-day course of azithromycin. After completing the course of medications, he experienced blurred vision in both eyes, headache, and eye pain that was exacerbated by extraocular movements. A day after symptom onset, he presented to an ophthalmologist. During this examination, his visual acuity was *count fingers* in the right eye (OD) and *hand motion* in the left eye (OS). The examination revealed optic disc edema and a white-centered retinal hemorrhage (Roth spot) in the mid-periphery OS. Optical coherence tomography (OCT) of the retinal nerve fiber layer (RNFL) showed an average RNFL thickness of 111 μm OD and 118 μm OS. Magnetic resonance imaging (MRI) of the brain, performed the morning after symptom onset, was interpreted as normal. MRI of the orbits was not included in the initial imaging studies.

**Figure 1:**
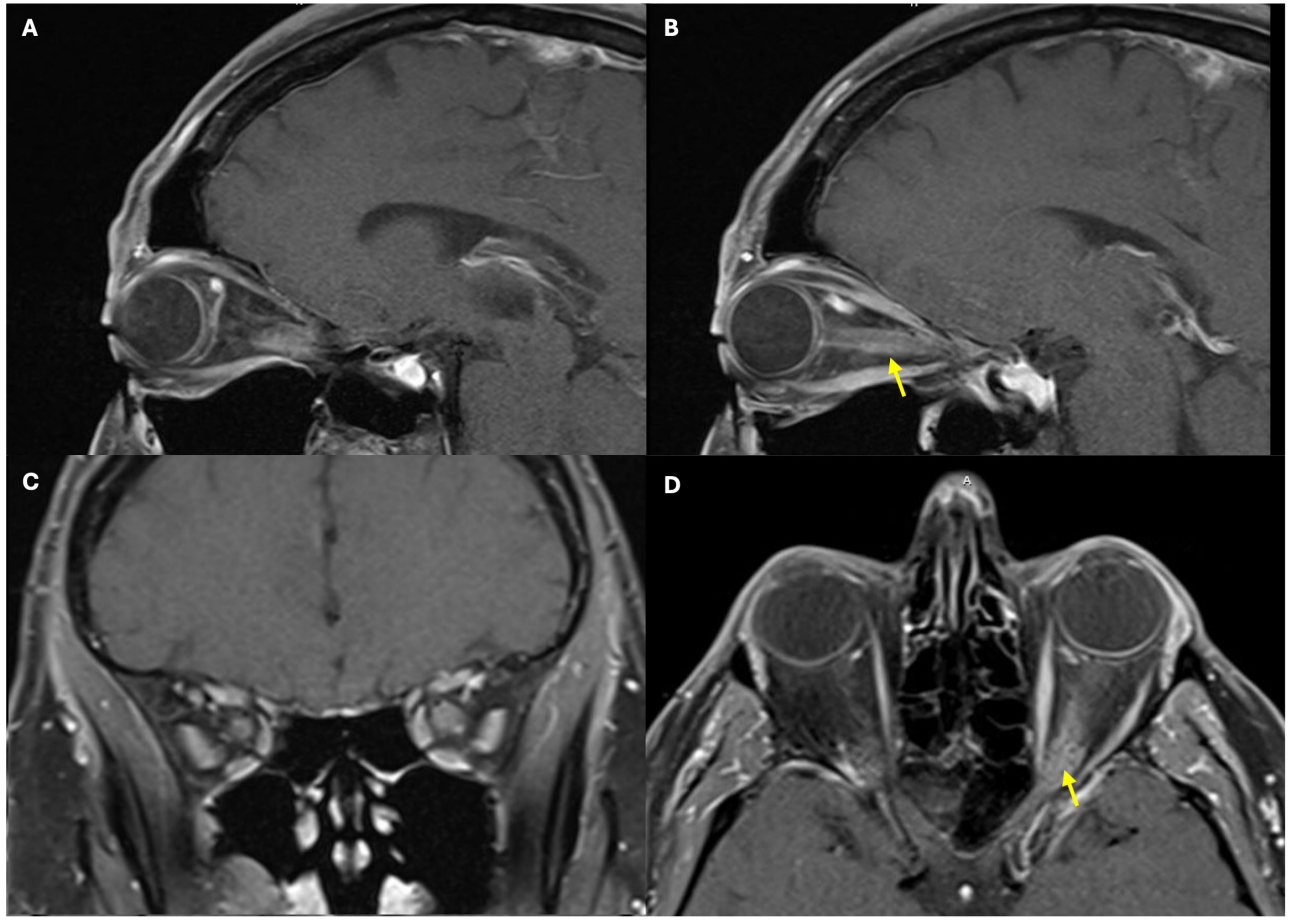
Magnetic resonance imaging of the brain and orbits demonstrating thickening and perineural enhancement along both optic nerves, the left greater than the right, with findings suggestive of optic neuritis. The figure includes sagittal views of the left optic nerve **(A, B)**, coronal view of the bilateral optic nerves **(C)**, and axial view of the bilateral optic nerves **(D)**. *Yellow arrows* highlight the significant thickening and perineural enhancement along the left optic nerve.

A day after his initial ophthalmology visit, the patient was admitted to an outside hospital. Repeat MRI of the brain and orbits revealed thickening and perineural enhancement along both optic nerves, which were more prominent on the left than on the right. These findings were suggestive of optic neuritis (**Figure 1**). He was started on broad-spectrum systemic antibiotics, acetazolamide 250 mg three times daily, and intravenous (IV) methylprednisolone 500 mg every 12 h. The patient was transferred to our facility 3 days later for further evaluation.

**Figure 2:**
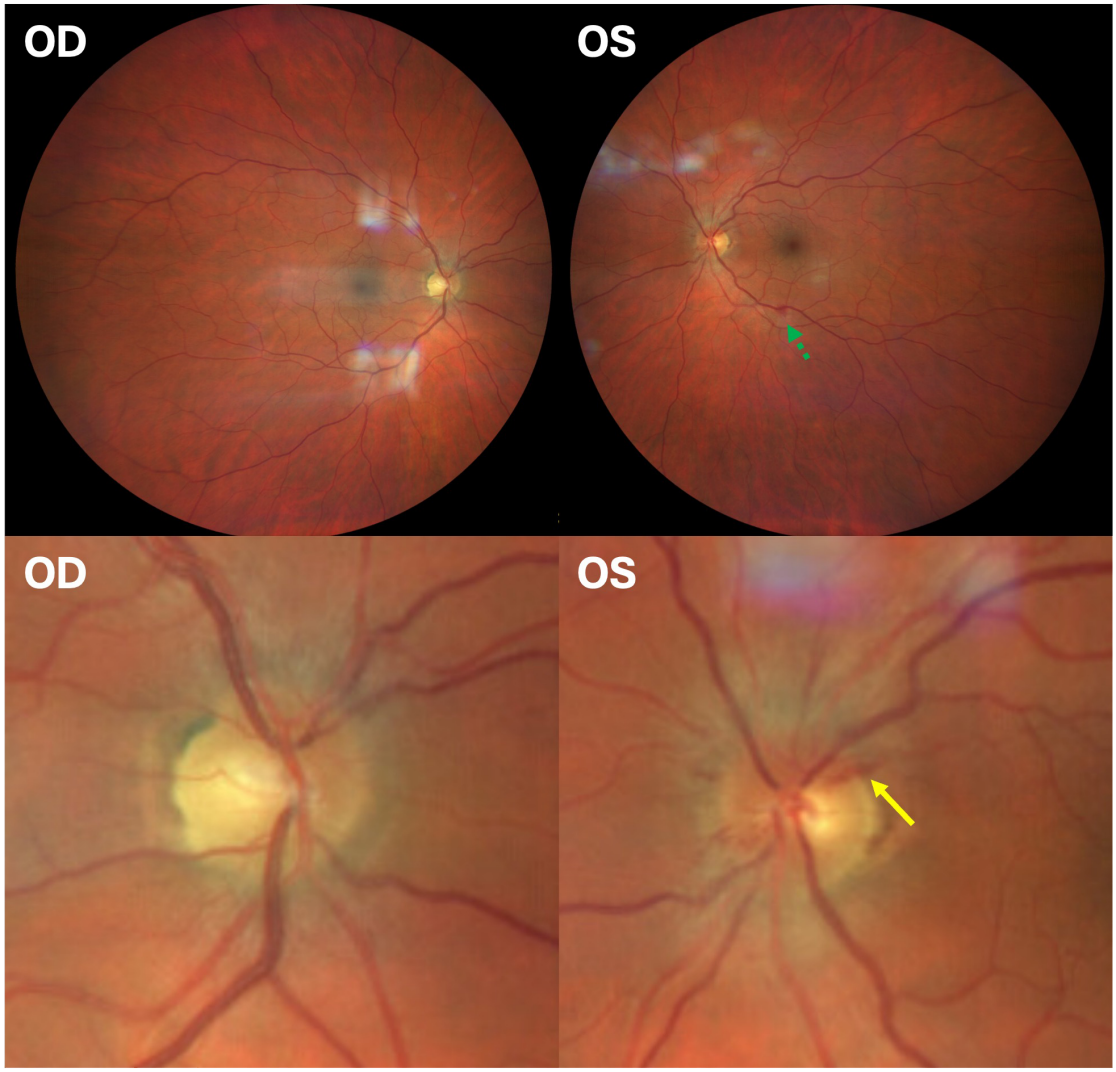
Fundus photographs of the right and the left eye. A retinal hemorrhage adjacent to an inferior retinal vein is noted by a *green dotted arrow*. The *lower images* show an enlarged view, demonstrating optic nerve edema and peripapillary hemorrhage (*yellow solid arrow*). The inferior and temporal Roth spots are not pictured in the left eye.

**Figure 3:**
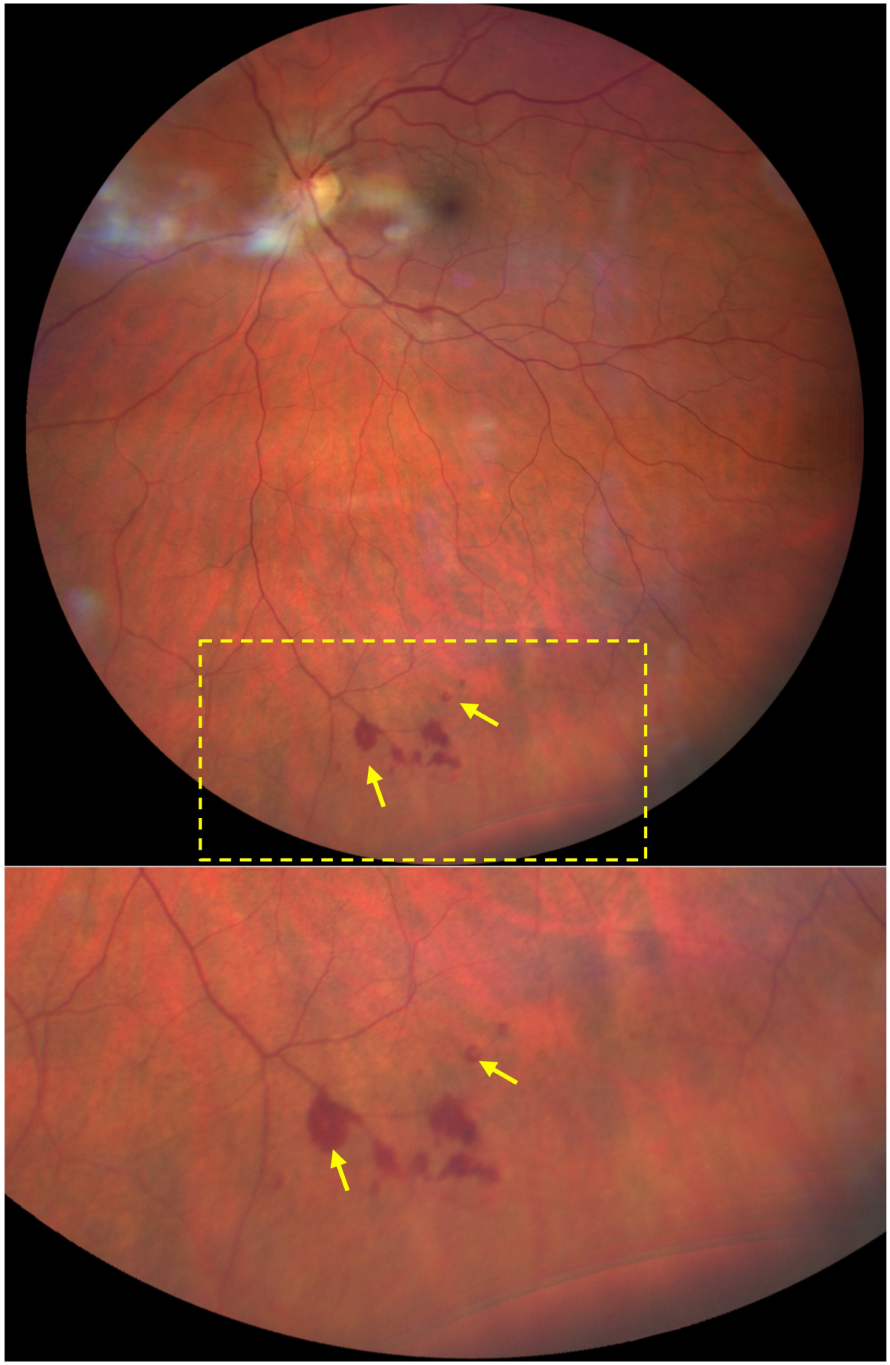
Fundus photograph of the left eye depicting intraretinal hemorrhages and Roth spots. Roth spots are highlighted with *yellow arrows*. The *lower image* shows an enlarged view of the concentrated hemorrhages along the inferior peripheral retina. Temporal Roth spot not pictured.

Upon admission, the patient reported subjective improvement in his vision and a reduction in pain during extraocular movements. Examination revealed a visual acuity of 20/25 OD and *count fingers* OS. A relative afferent pupillary defect was present in the left eye. The anterior segment appeared unremarkable in both eyes (OU). The patient was unable to read any Ishihara color plate, including the control plate, using either eye. Fundoscopy showed bilateral optic disc edema that was worse OS. Peripapillary hemorrhages were identified OS (**Figure 2**). Several intraretinal hemorrhages and Roth spots were located inferiorly (**Figure 3**) and temporally.

The patient underwent an extensive workup. Notable laboratory findings included a positive MOG immunoglobulin G (IgG) antibody with a serum titer of 1:1,000, which was obtained via fluorescence-activated cell sorting. The initial complete blood count and the complete metabolic panel were unremarkable (**Table 1**). The serum antinuclear antibody, extractable nuclear antigen, and anti-neutrophilic cytoplasmic antibody tests were negative. The erythrocyte sedimentation rate and the C-reactive protein levels were normal. Further serum laboratory testing for human immunodeficiency virus, tuberculosis, syphilis, *Bartonella*, Lyme disease, and *Toxoplasma gondii* was negative. His vitamin D levels were low at 18 ng/mL. Transthoracic echocardiogram showed no evidence of endocarditis. Magnetic resonance angiography and venography of the brain, as well as MRI of the spine, were normal. Cerebrospinal fluid (CSF) analysis showed an elevated nucleated cell count (31/mm^3^) with 95% lymphocytes and an elevated CD8 count (894 cells/μL). CSF studies including cryptococcal antigen, *T. gondii* polymerase chain reaction, bacterial culture, and fungal culture were negative. CSF analysis revealed no oligoclonal bands and no significant B-cell population. Opening pressure on lumbar puncture was not recorded.

**Table 1:** Laboratory evaluation.

Complete Blood Count	
White blood cell count	13.7 K/cumm (high)
Hemoglobin	14.5 g/dL
Hematocrit	45.8%
Platelet	233 K/cumm
Comprehensive Metabolic Panel	
Sodium	144 mmol/L
Potassium	3.8 mmol/L
Chloride	109 mmol/L
Bicarbonate	20 mmol/L (low)
Anion gap	15 mmol/L
Blood urea nitrogen	24 mg/dL
Creatinine	0.93 mg/dL
Glucose	120 mg/dL
Calcium	9.1 mg/dL
Magnesium	2.5 mg/dL
Phosphorous	2.9 mg/dL
Bilirubin, total	0.5 mg/dL
Protein	7.0 g/dL
Albumin	4.2 g/dL
Estimated glomerular filtration rate	>90 mL/min/1.73 m^2^
Alkaline phosphatase	68 Units/L
Aspartate aminotransferase	25 Units/L
Alanine aminotransferase	30 Units/L
Additional Serum Laboratory Evaluation	
Antinuclear antibody	Negative
Extractable nuclear antigen	Negative
Anti-neutrophilic cytoplasmic antibody	Negative
Erythrocyte sedimentation rate	8 mm/hr
C-reactive protein	0.6 mg/L
Human immunodeficiency virus antibody + p24 antigen	Nonreactive
Rapid plasma reagin	Nonreactive
Treponemal IgG/IgM	Nonreactive
T-SPOT.TB	Negative
Bartonella henselae, IgM	<1:128
Bartonella henselae, IgG	<1:20
Lyme antibody	Negative
Toxoplasma IgM	Negative
Toxoplasma IgG	Negative
Vitamin D 25-OH	18 ng/mL (low)

IgM, Immunoglobulin M; IgG, Immunoglobulin G.

**Figure 4:**
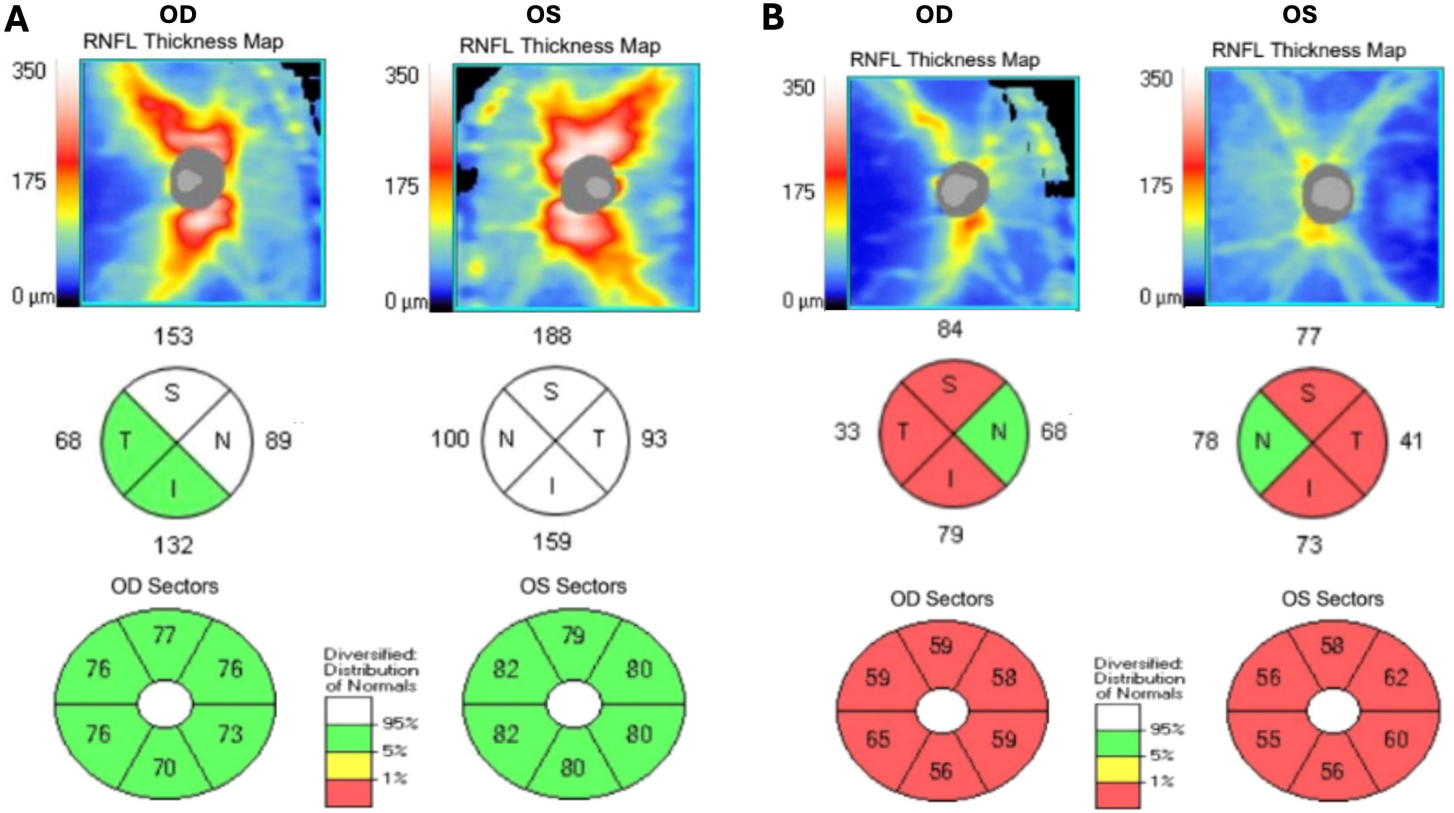
Spectral domain optical coherence tomography demonstrating retinal nerve fiber layer thickening in two quadrants in the right eye and all four quadrants in the left eye on initial presentation **(A)**, with subsequent retinal nerve fiber layer and ganglion cell complex thinning at 8-month follow-up **(B)**.

Positron emission tomography/computed tomography (PET/CT) showed mild ^18^F-fludeoxyglucose uptake along the optic nerves, consistent with optic neuritis. Whole-body PET/CT imaging and CT imaging of the chest, abdomen, and pelvis were negative for occult malignancy. The patient underwent repeat OCT of the RNFL, which showed stable RNFL thickness in the right eye (111 μm) and an increased RNFL thickness in the left eye (135 μm). OCT of the macula was normal, and analysis of the ganglion cell complex (GCC) revealed normal average thickness in both eyes: 75 μm OD and 80 μm OS (**Figure 4**).

During his admission, he was treated with an additional 3 days of 1 g of IV methylprednisolone daily, followed by an oral prednisone taper. The patient had a gradual recovery of vision in the left eye and was subsequently treated with five sessions of plasmapheresis. After completion of the plasmapheresis regimen, he showed improvement in both subjective symptoms and objective visual acuity. The patient was subsequently discharged with a visual acuity of 20/20 OD and 20/30 OS. Repeat fundoscopy at discharge revealed stable intraretinal hemorrhages and Roth spots in the temporal and inferior posterior pole OS.

At 6 weeks after hospital discharge, the patient was evaluated in the outpatient setting. His visual acuity was 20/20 OU. The patient had a persistent relative afferent pupillary defect OS. OCT revealed RNFL thickness of 82 μm OD and 87 μm OS, while OCT of the GCC showed mild bilateral thinning, measuring 64 μm OD and 65 μm OS. Fundoscopy revealed resolution of the previously identified retinal hemorrhages and Roth spots. At an outpatient visit 3 months after the initial hospitalization, the patient reported a mild recurrence of symptoms, including color vision abnormalities and increased light sensitivity, following a recent decrease in prednisone dosage. Due to the continued symptoms, the patient’s steroid taper was slowed. Despite continued symptoms, repeat testing at the 3-month follow-up visit showed a decrease in the serum MOG IgG titer to 1:100, down from the initial 1:1,000 at admission. At the 8-month follow-up appointment, the patient had finished the prednisone taper and was largely symptom-free. OCT revealed progressive thinning of the RNFL (66 μm OD and 67 μm OS) and the GCC (59 μm OD and 58 μm OS) (**Figure 4**).

## 3 Discussion

Retinal hemorrhages and Roth spots have not traditionally been associated with MOG-ON, and their presence may obscure or delay diagnosis. We presented a rare case of MOG-ON with unilateral retinal hemorrhages and Roth spots, expanding the clinical spectrum of fundus findings linked to this condition.

The diagnosis of MOG-ON in this case was supported by imaging that demonstrated perineural enhancement along the optic nerves, a finding highly suggestive of an inflammatory etiology (1). The patient had an elevated serum MOG IgG titer. His clinical course included a modest improvement in vision after high-dose steroids, followed by near-complete recovery in visual acuity after plasmapheresis, which is consistent with a typical recovery trajectory of MOG-ON. While retinal hemorrhages and Roth spots are nonspecific findings seen in infectious, inflammatory, and oncologic conditions, the patient’s well-controlled systemic diseases and unrevealing systemic workup argue against alternative etiologies. Moreover, the resolution of the intraretinal hemorrhages and Roth spots following treatment, along with the absence of retinal hemorrhages in the less affected contralateral eye, provides both spatial and temporal support for an association with the patient’s MOG-ON.

To our knowledge, this is only the third case of MOG-ON with concurrent retinal hemorrhages and Roth spots. Until recently, neither retinal hemorrhages nor Roth spots have been recognized features of MOG-ON, and fundus findings beyond optic nerve edema and peripapillary hemorrhages are rare. Retinal hemorrhages in MOG-ON may be underreported in the literature given their lack of specificity. A PubMed literature review identifying cases of MOG-ON associated with retinal hemorrhages or Roth spots before 30 March 2025 yielded 107 results. Among these, only nine cases (8.4%) documented hemorrhages outside of the peripapillary region. Notably, five of these nine cases had systemic comorbidities, including diabetes, hypertension, and coronavirus disease 2019 (COVID-19) (**Table 2**). Only two cases (1.8%) documented Roth spots in patients with MOG-ON, both in female patients aged 27 and 49 years, respectively (2, 3). Similar to these reports, the presentation of our patient prompted an extensive systemic evaluation, underscoring the diagnostic challenge posed by the fundoscopic findings.

**Table 2:** Initial episodes of MOG-ON - clinical features and fundus findings beyond optic disc edema and peripapillary hemorrhage.

Case Report	Age, Sex	BCVA (OD, OS)	Fundus Findings	Pertinent Medical History	Presenting Ocular Complaint	Serum MOG Titer	Treatment	Follow Up
Zhou 2020 (10)	26, M	HM, 20/250	Bilateral disc edema, venous congestion, retinal perivenous hemorrhages OD	+ SARS-CoV-2 PCR	Presented with painful vision loss of the left eye followed by the right eye	1:1000	IV Methylprednisolone 1 g daily for 5 days followed by oral prednisone taper	Significant VA recovery
Mittal 2021 (11)	32, F	20/20, CF	Left optic disc edema with scattered intraretinal and subretinal hemorrhages throughout the posterior pole without vascular sheathing or vitritis	Hypertension, Diabetes Mellitus	Presented with 3 days of vision loss and painful eye movements following headache	1:100	IV Methylprednisolone 1 g daily for 3 days, then 100 mg oral prednisone for 1 week with a 4-day taper by 20 mg each day	Full VA recovery, Thought to be initial presentation of Sjogren’s disease
Aboab 2022 (3)	49, F	20/15, CF	Severe optic disc edema with multiple peripapillary hemorrhages, peripheral retinal hemorrhages, and Roth spots OS	N/A	Presented with painful vision loss OS	Positive	IV Methylprednisolone 1 g daily for 6 days, followed by 6 months of oral steroid taper. Oral azathioprine for immunosuppression	Full VA recovery
Lukewich 2020 (12)	38, F	LP, 20/20	Severe optic disc edema with optic disc hemorrhages, dilated and tortuous retinal venules, and small dot hemorrhages 360° throughout the retina without macular edema OD	Hypertension	Presented with 2 weeks of vision loss and pain with eye movements	Positive	IV Methylprednisolone 1 g daily for 5 days followed by oral prednisone taper. Plasmapheresis for 7 days. Azathioprine for long-term immunosuppression.	Full VA recovery, significant RNFL and GCC thinning OD
Schichtel 2024 (13)	57, M	20/150, HM	3+ optic disc edema with retinal nerve fiber layer hemorrhages and cotton wool spots/ischemia OU	Hypertension, Diabetes Mellitus	Presented with vision loss	1:1000	IV Methylprednisolone 500 g twice daily for 5 days followed by plasmapheresis for 5 cycles.	Partial VA recovery
Srimanan 2024 (2)	27, F	20/800, 20/20	Preretinal hemorrhage measuring approximately 10-disc diameters at the posterior pole involving the macular area with vitreous hemorrhage, optic disc edema with hyperemia, scattered intraretinal hemorrhage, Roth spots, and tortuous, dilated veins without sheathing vessels	N/A	Presented after 2 weeks of worsening headache	Positive	IV Methylprednisolone 1 g daily for 5 days followed by oral prednisone taper	Partial VA recovery
Teru 2024 (14)	64, M	“Vision loss”	Grade 4 disc edema and significant burden of dot blot hemorrhages in all quadrants of the right eye	Hypertension, Factor V Leiden, Recent COVID-19 infection	Presented with 10 days of progressive right eye vision loss and headache	1:100	IV Methylprednisolone and plasmapheresis followed by maintenance rituximab treatment.	Partial VA recovery
Wilke 2024 (4)	15, F	20/20, LP	Grade III optic disc edema OD and grade IV optic disc edema OS with peripapillary hemorrhages OU. Retinal vascular tortuosity and small intraretinal hemorrhages in the macula and periphery OU	N/A	Presented with headaches and retroorbital “eye pressure”	1:100	Plasmapheresis for 6 days followed by 5-week oral prednisone taper	Full VA recovery
Li 2024 (15)	39, F	NLP, NLP	Bilateral peripapillary and perivascular hemorrhages as well as extensive deep retinal hemorrhages with severe optic disc edema, OD>OS; dilated and tortuous vessels	+ EBV Antigen (595 units/mL) and + EBV capsid antigen (>750 units/mL)	Presented with four days of vision loss	1:320	IV Methylprednisolone 1 g daily for 6 days and plasma exchange followed by oral prednisolone taper. Rituximab twice weekly every other week	Full VA recovery

CF, count fingers; GCC, ganglion cell complex; HM, hand motion; LP, light perception; MOG, myelin oligodendrocyte glycoprotein; MOG-ON, myelin oligodendrocyte glycoprotein associated optic neuritis; NLP, no light perception; OD, right eye; OS, left eye; RNFL, retinal nerve fiber layer; VA, visual acuity

The etiology of retinal hemorrhages and Roth spots in MOG-ON remains largely unknown, although several mechanisms have been proposed. One proposed mechanism is impaired retinal venous outflow secondary to optic disc edema, as supported by previous reports (2–4). In this case, the patient demonstrated perineural enhancement on MRI, raising the possibility of vascular occlusion as a rare downstream consequence of optic nerve sheath involvement, as seen in optic perineuritis (5). These mechanisms suggest vascular congestion as a contributing factor rather than a direct implication of the pathogenesis of MOG antibody-associated disease (MOGAD). Notably, MOGAD-related inflammation involves a complex interplay between humoral and cellular immune responses (6, 7). Perivascular inflammation has been observed in MOGAD and shows phenotypic and pathologic overlap with central nervous system vasculitis (8, 9). Thus, vascular inflammation may underlie the development of Roth spots in this patient. Although fluorescein angiography was not performed, it might have provided additional evidence to clarify the mechanism for the retinal findings. OCT did not reveal evidence of intraretinal inflammation.

## 4 Conclusions

The final diagnosis was MOG-ON with associated intraretinal hemorrhages and Roth spots. The diagnosis of MOG-ON was supported by a classic clinical course, characteristic imaging findings, and a high MOG IgG serum titer. While earlier descriptions of MOG-ON fundus findings highlighted optic nerve edema and peripapillary hemorrhage, retinal hemorrhages have now emerged as a documented clinical finding in more recent reports. To our knowledge, this is the first reported case of retinal hemorrhages and Roth spots associated with MOG-ON in a male patient, and only the third reported case of any patient with these findings. This case helps to better differentiate the fundus findings associated with MOG-ON, identify intricacies in its presentation, and aid in the recognition of a disease that can have potentially devastating ocular sequelae.

## Data Availability

The original contributions presented in the study are included in the article/supplementary material, further inquiries can be directed to the corresponding author/s.
